# Target Fortification of Breast Milk: Predicting the Final Osmolality of the Feeds

**DOI:** 10.1371/journal.pone.0148941

**Published:** 2016-02-10

**Authors:** Arum Choi, Gerhard Fusch, Niels Rochow, Christoph Fusch

**Affiliations:** Division of Neonatology, Department of Pediatrics, McMaster University, Hamilton, Ontario, Canada; TNO, NETHERLANDS

## Abstract

For preterm infants, it is common practice to add human milk fortifiers to native breast milk to enhance protein and calorie supply because the growth rates and nutritional requirements of preterm infants are considerably higher than those of term infants. However, macronutrient intake may still be inadequate because the composition of native breast milk has individual inter- and intra-sample variation. Target fortification (TFO) of breast milk is a new nutritional regime aiming to reduce such variations by individually measuring and adding deficient macronutrients. Added TFO components contribute to the final osmolality of milk feeds. It is important to predict the final osmolality of TFO breast milk to ensure current osmolality recommendations are followed to minimize feeding intolerance and necrotizing enterocolitis. This study aims to develop and validate equations to predict the osmolality of TFO milk batches. To establish prediction models, the osmolalities of either native or supplemented breast milk with known amounts of fat, protein, and carbohydrates were analyzed. To validate prediction models, the osmolalities of each macronutrient and combinations of macronutrients were measured in an independent sample set. Additionally, osmolality was measured in TFO milk samples obtained from a previous clinical study and compared with predicted osmolality using the prediction equations. Following the addition of 1 g of carbohydrates (glucose polymer), 1 g of hydrolyzed protein, or 1 g of whey protein per 100 mL breast milk, the average increase in osmolality was 20, 38, and 4 mOsm/kg respectively. Adding fat decreased osmolality only marginally due to dilution effect. Measured and predicted osmolality of combinations of macronutrients as well as single macronutrient (R^2^ = 0.93) were highly correlated. Using clinical data (n = 696), the average difference between the measured and predicted osmolality was 3 ± 11 mOsm/kg and was not statistically significant. In conclusion, the prediction model can be utilized to estimate osmolality values after fortification.

## Introduction

Breast milk is the best source of nutrition for preterm infants but does not alone provide optimal nutrition. Standard fortification of breast milk is defined as breast milk with the addition of commercially available human milk fortifier. It is routine practice in Neonatal Intensive Care Units (NICUs) to adapt native breast milk to the high dietary needs of these fast growing infants. However, due to inter- and intra-individual macronutrient variations, standard fortified breast milk may still fall short of recommended macronutrient intakes for some preterm infants [1–3]. The concept of target fortification has been developed to add deficient macronutrients to each fortified breast milk sample after an analysis of macronutrient components in native breast milk [3]. This fulfills the recommended nutritional requirements for each infant by making up for macronutrient variations in standard fortified breast milk according to international guidelines [4–5]. Hence, target fortification is a means to overcome nutritional deficit and reduces the risk for postnatal growth restriction [3]. In a recently published study it was demonstrated that infants who were fed target fortified breast milk showed a significant correlation between weight gain and volume of milk intake (R^2^ = 0.68; p = 0.004) whereas those on standard fortification did not (R^2^ = 0.02; p = 0.58) [3]. The downside of target fortification is a mild increase in osmolality of feeds due to added macronutrient components. In particular, additives containing glucose-polymer can significantly increase osmolality [6]. It has been reported that the high osmolality of fortified breast milk can lead to delayed gastric emptying and reduced intestinal peristalsis [7]. Currently, an upper limit of 450 mOsm/kg (i.e. 400 mOsm/L) has been suggested for enteral feedings though this recommendation is not based on solid scientific evidence [8–9].

In our recent study, the standard fortification with human milk fortifier such as Similac (Ross products division, Abbott Nutrition, Columbus, USA) enhances the osmolality of native breast milk from 298 ± 7 mOsm/kg to 405 ± 14 mOsm/kg (data are not shown) [3]. Target fortification that meets international nutrient guidelines further increases osmolality up to 436 ± 13 mOsm/kg without inducing feeding intolerance and is within normal ranges of biochemical safety variables [3]. Furthermore, clinical routines like sample storage have been identified as a factor that contributes to the final osmolality of milk feeds. It has been shown that osmolality had risen by 2.5–5.0% within 10 minutes after standard fortification, mostly because of the breakdown of carbohydrates [10]. A further 4% increase in osmolality was seen after storage at 4°C for 24 hours [11]. Therefore, evaluating the osmolality of target fortified breast milk is necessary to ensure safe feeds, especially for very low birth weight preterm infants.

The aims of the study are (1) to establish and validate prediction models for the osmolality of breast milk after adding macronutrients, (2) to validate the prediction models using target fortified breast milk samples obtained from a clinical study and (3) to investigate whether breast milk storage (4°C, 24h) affects osmolality. We hypothesized that the final osmolality of target fortified breast milk samples can be predicted.

## Materials and Methods

For this study, leftover breast milk samples (n = 40) each obtained during a full lactation cycle (fore, mid, hind milk) were donated from a single mother in the NICU at McMaster Children’s Hospital (Hamilton, Ontario, Canada). Oral consent was obtained prior to sample collection and later completed in written form. The prediction model was validated using data from our previous study (approved by the Hamilton Integrated Research Ethics Board (HIREB) of McMaster University, HIREB—#12–109) [3]. The experimental part of the study was also presented to the HIREB and approved as a quality improvement study. All samples were stored at -20°C until analysis. To test the impact of components used for target fortification, commercially available macronutrient products were used: Polycose (glucose polymer) (Abbott Nutrition, Columbus, USA) for carbohydrates, Beneprotein (whey protein) (Protein 1, Nestle HealthCare Nutrition, Minneapolis, USA) and Aptamil Protein (1:1 whey/casein hydrolyzed protein) (Protein 2, Milupa, Lisbon, Portugal) for protein, and Microlipid (Nestle HealthCare Nutrition, Minneapolis, USA) for fat. The following two commercially available milk fortifiers were investigated; HMF1: Similac human milk fortifier (Ross Products Division, Abbott Nutrition, Columbus, USA) and HMF2: Enfamil human milk fortifier (Mead Johnson & Company, Evansville, USA).

For preparing standard fortified breast milk samples, one pack of human milk fortifier was used for every 25 mL of breast milk. All additives were weighed to the nearest 0.1 mg using an analytical balance (Mettler AT261, Greifensee, Switzerland). All samples were prepared as 25 ml volumes and data were converted per 100 mL of breast milk.

To ensure a representative aliquot of breast milk was drawn, breast milk samples (volume of 25 mL) were homogenized for 30 seconds using an ultrasonic vibrator (VCX 130, Chemical Instruments AB, Sollentuna, Sweden). The level of osmolality was determined on native breast milk and after each fortification step using freezing point depression (3320 Micro-Osmometer, Advanced Instruments Inc., Norwood, MA, USA). The device was calibrated with calibration standards (Advanced Instruments Inc, Norwood, MA, USA). Quality control measurements (0.9% sodium chloride, Hospitra, Montreal, QC, CA) were conducted after every 10 measurements.

### 1) Establishment of osmolality prediction models for each macronutrient

Native breast milk (250 mL) was pooled and homogenized for 3 minutes. The milk was divided into 10 x 25 mL. Each macronutrient was then gradually added to 25 mL homogenized breast milk to cover the variation of macronutrients and to reflect clinical practice of target fortification of breast milk [3]. For carbohydrates, the initial concentration of prepared breast milk was 0.125 g/25 mL. Carbohydrates were added to produce final concentrations of 0.25 g/25 mL, 0.375 g/25 mL, and 0.5 g/25 mL. For protein, the initial concentration was 0.05 g/25 mL. Protein was added gradually to reach the final concentrations of 0.125 g/25 mL, 0.25 g/25 mL, 0.5 g/25 mL. For fat, the initial concentration was 0.125 mL/25 mL. Fat was added gradually to reach the final concentrations of 0.25 mL/25 mL, 0.5 mL/25 mL, and 1.0 mL/25 mL. For each increment step, the sample was again homogenized for 30 seconds to ensure milk fat globules were dispersed uniformly throughout the milk [12] and osmolality of the sample was immediately measured. Osmolality was measured after each enrichment step (S1 Table).

### 2) Validation of osmolality prediction models for each macronutrient

To validate the prediction model, two test solutions, one with a low concentration and one with a high concentration, but both within the ranges usually expected in clinical routine were prepared for each additive. Each concentration step of each macronutrient was done in 25 mL of homogenized native breast milk (n = 5 for each additive) and was again homogenized for 30 seconds. Osmolality was measured immediately after fortification (S2 Table).

### 3) Validation of prediction models using more complex combinations of three additives

To simulate target fortification of breast milk, procedures were performed as previously described in our pilot trial on target fortification [3]. Thawed native breast milk batches (n = 10, each aliquot of 25 mL) were homogenized and macronutrient composition was analyzed using a validated milk analyzer (Unity Scientific, Brookfield, CT, USA) [12]. Standard fortification was performed using HMF1 and afterwards supplemental amounts of fat, protein, and carbohydrates were added to reach target fortification levels [4]. All four combinations that can potentially be achieved by adding fat, protein, and carbohydrates (i.e. fat + protein, fat + carbohydrates, protein + carbohydrates, fat + protein + carbohydrates) were assessed. After fortification, samples were homogenized for 30 seconds and osmolality of the samples were measured. These measured osmolality values were compared with predicted osmolality using the prediction models (S3 Table).

### 4) Osmolar contribution of different human milk fortifiers

Native breast milk batches (n = 10, each aliquot of 25 mL) were fortified with HMF1 and homogenized for 30 seconds. The same was done using HMF2. Osmolality values of these samples were immediately measured.

### 5) Change in osmolality after 24 hours storage at 4°C

To evaluate the impact of storage time on osmolality batches fortified with carbohydrates as described in section #1, these samples were stored for 24 hours at 4°C, which is in line with standard breast milk storage guidelines [13]. Osmolality values of these samples were determined and differences before and after storage were evaluated (S4 Table).

### 6) Comparison between predicted osmolality data and clinical data from the target fortification pilot study

To check the feasibility of the prediction model in clinical routine, osmolality data (n = 696) as well as their corresponding macronutrient contents (i.e. fat, protein, carbohydrates levels) were obtained from our recent study on target fortification [3]. The final osmolality values were predicted based on the measured osmolality of the corresponding native breast milk, the added amounts of fat, protein, and carbohydrates using the prediction model and by adding the osmolality value for HMF1 (see results: 106 mOsm/kg) [3]. The predicted values were compared to the measured osmolality values (S5 Table).

### Statistical analysis

Linear regression analysis on prediction models and the validation of these prediction models were performed using Microsoft Excel 2010 (Redmond, WA, USA).

## Results

**Fig 1:**
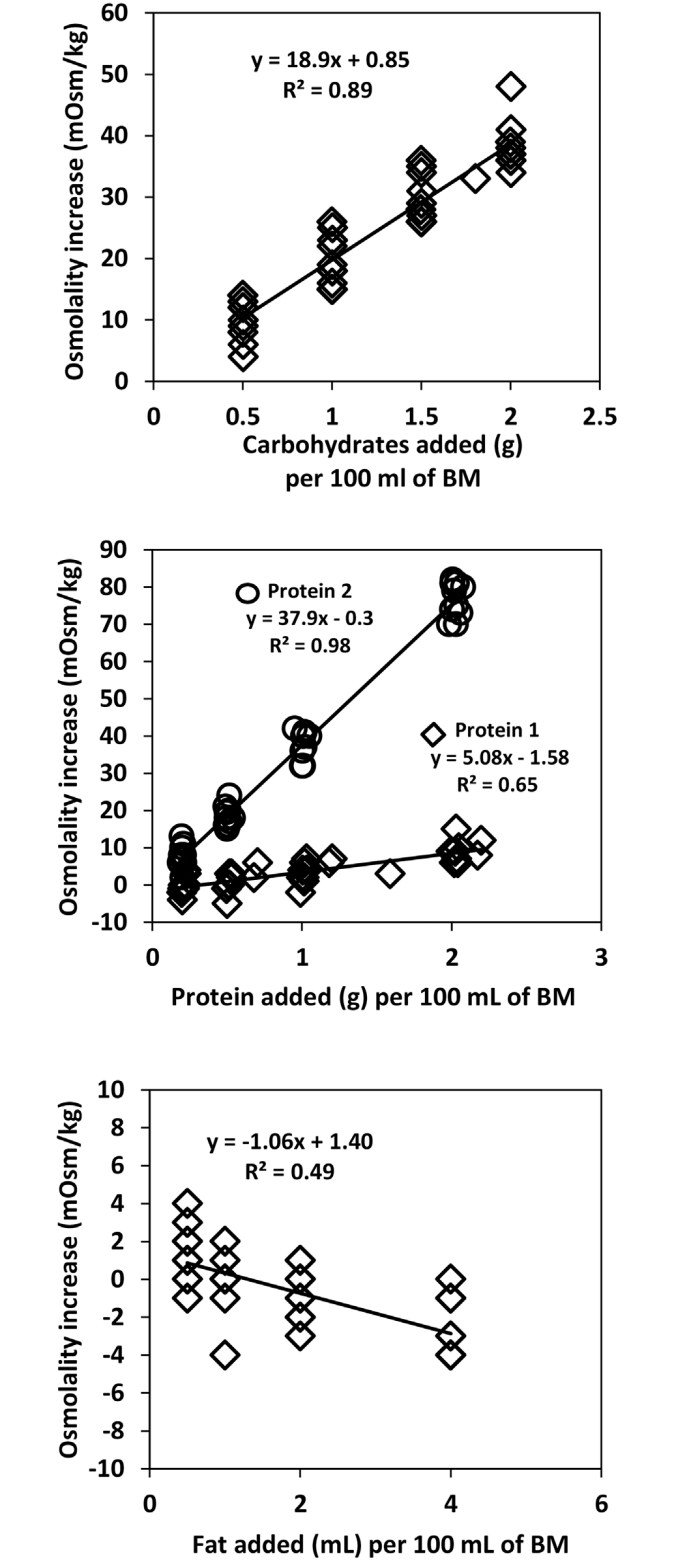
Prediction: linear correlation between increase in osmolality and added amount of each macronutrient in 100 mL of breast milk. Graphs on carbohydrates (glucose polymer), protein 1(whey protein), protein 2 (hydrolyzed protein), and fat from top to bottom.

**Fig 2:**
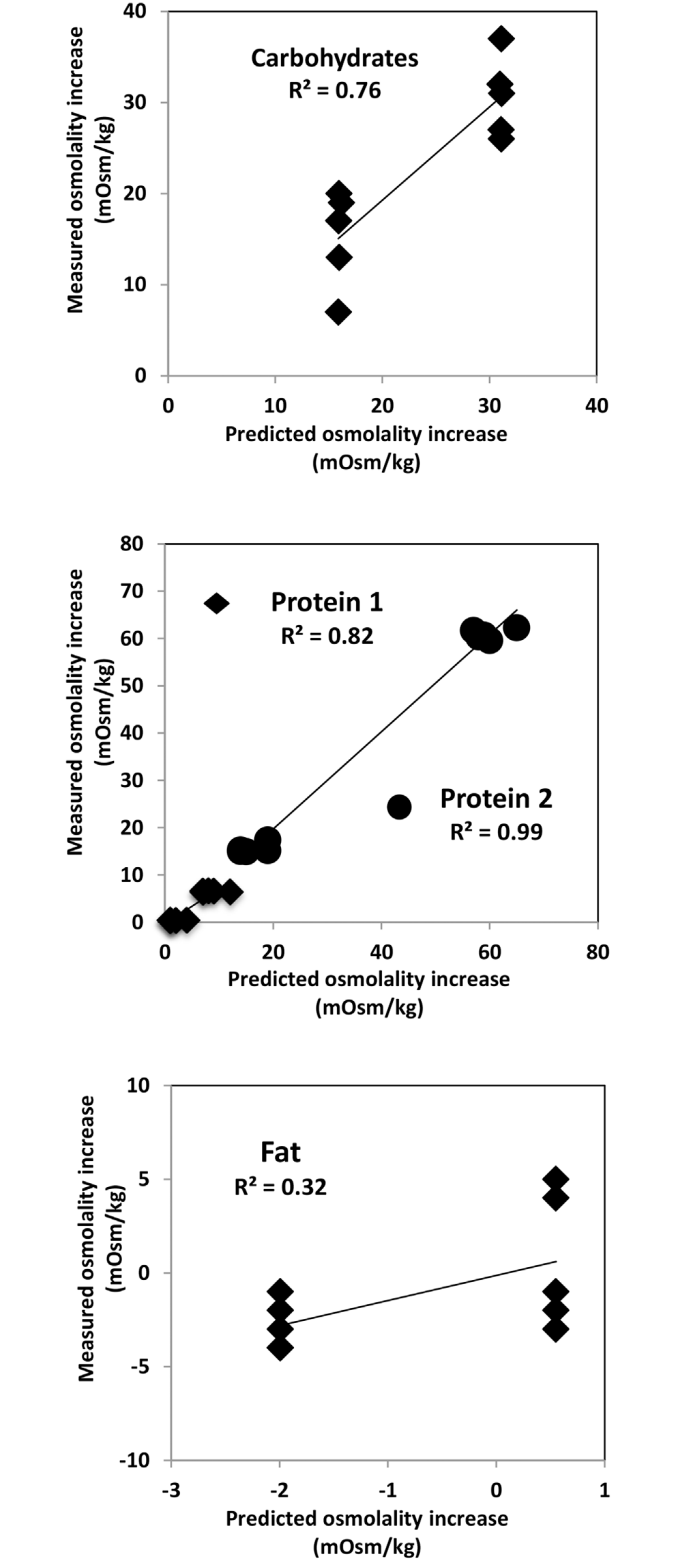
Validation 1: correlation between measured osmolality increase (i.e. measurements using a freezing point device) and predicted osmolality increase (i.e. calculations from the prediction equations) on single macronutrient. Graphs on carbohydrates (glucose polymer), protein 1 (whey protein), protein 2 (hydrolyzed protein), and fat from top to bottom.

**Fig 3:**
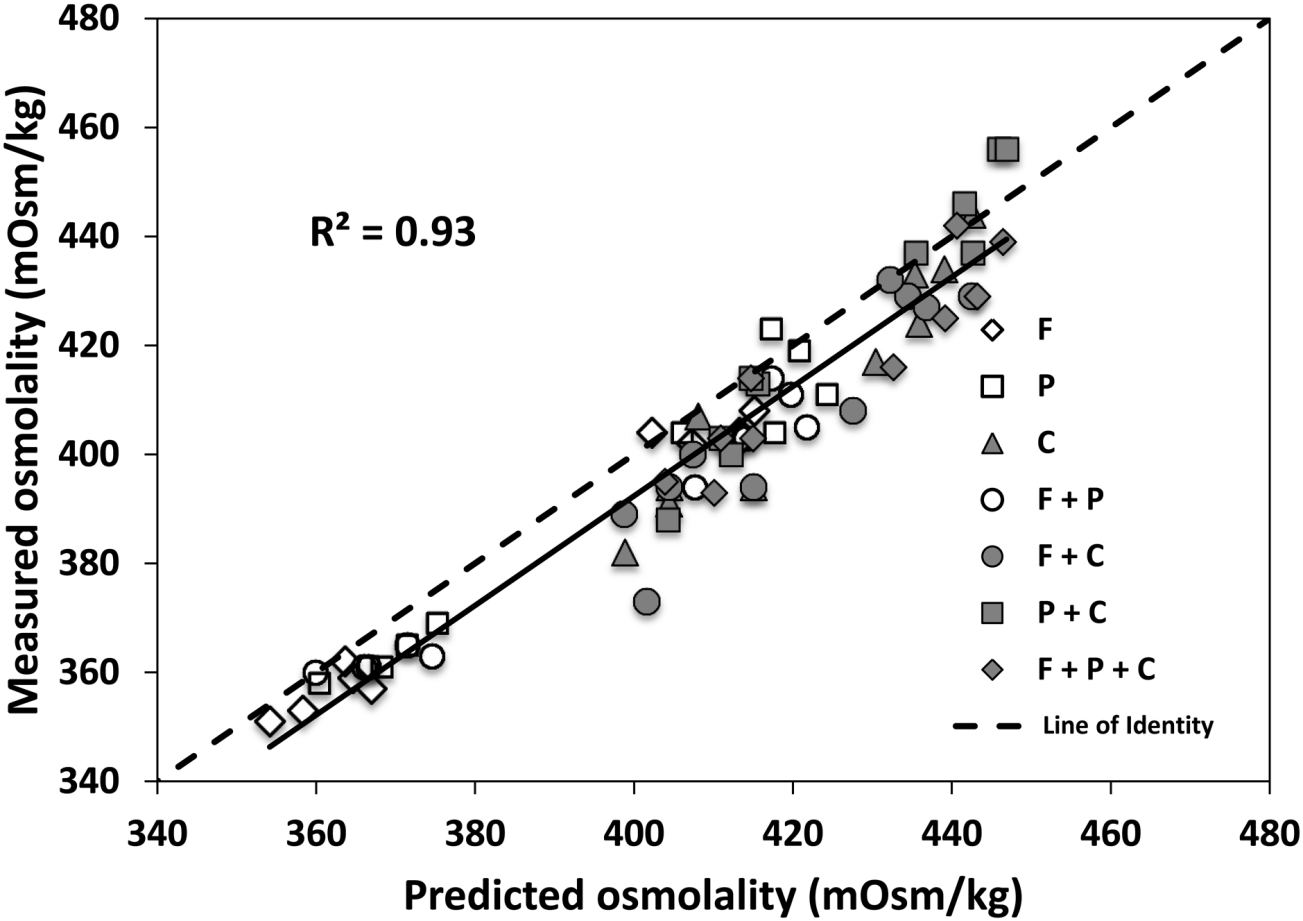
Validation 2: correlation between measured (i.e. measurements using a freezing point device) and predicted (i.e. calculations from the prediction equations) osmolality of breast milk with added combinations of macronutrients (Fat: F, Whey Protein: P, Glucose Polymer Carbohydrates: C). The average quantity to fortify in 100 mL breast milk was 1.3 mL (0.0, 3.2) (min, max), 1.2 g (0.6, 1.5), 1.8 g (1.1, 2.7) for F, P, C, respectively.

**Fig 4:**
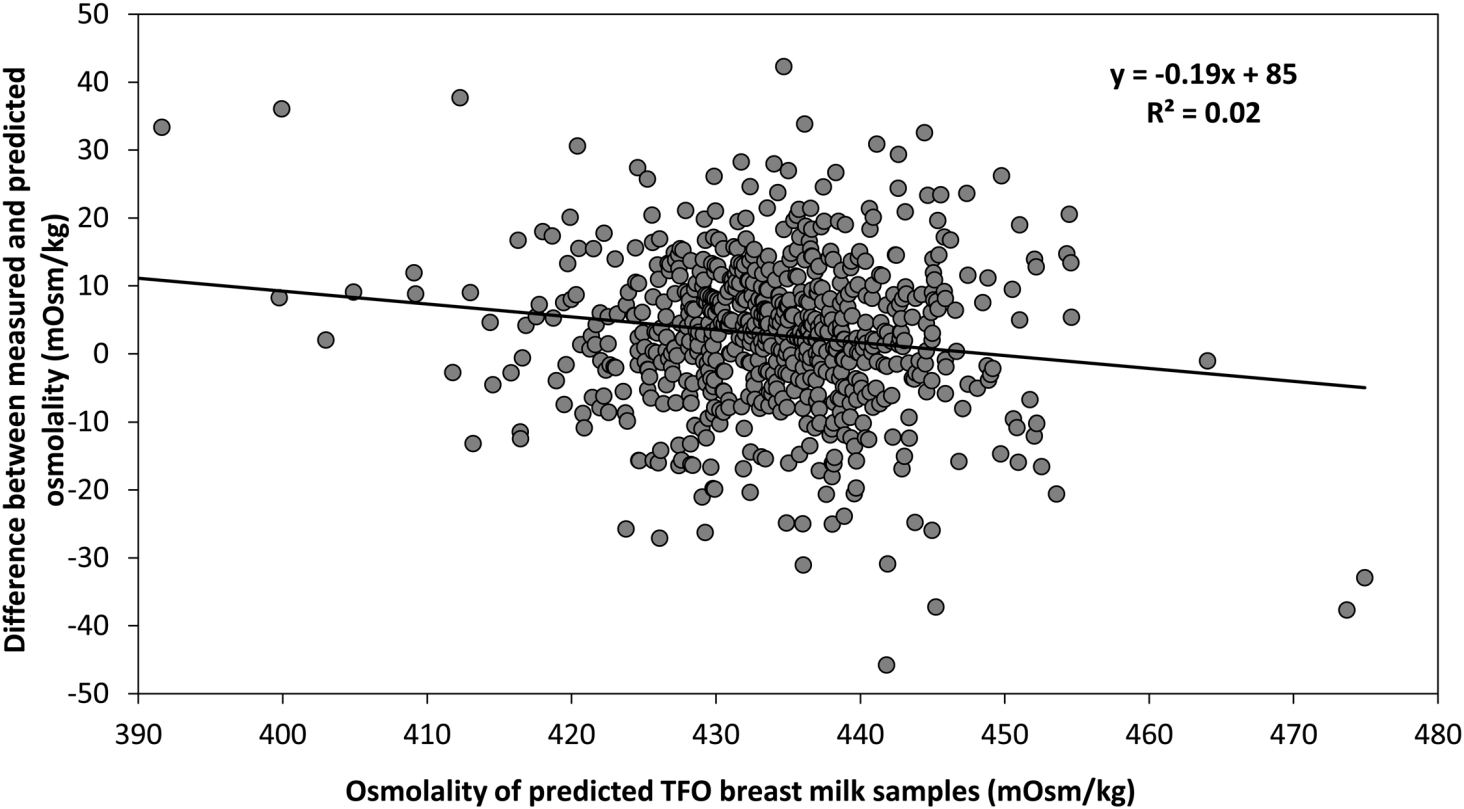
Deviation between the measured and predicted osmolality, using clinical samples from the pilot target fortification (TFO) study (n = 696).

**Fig 5:**
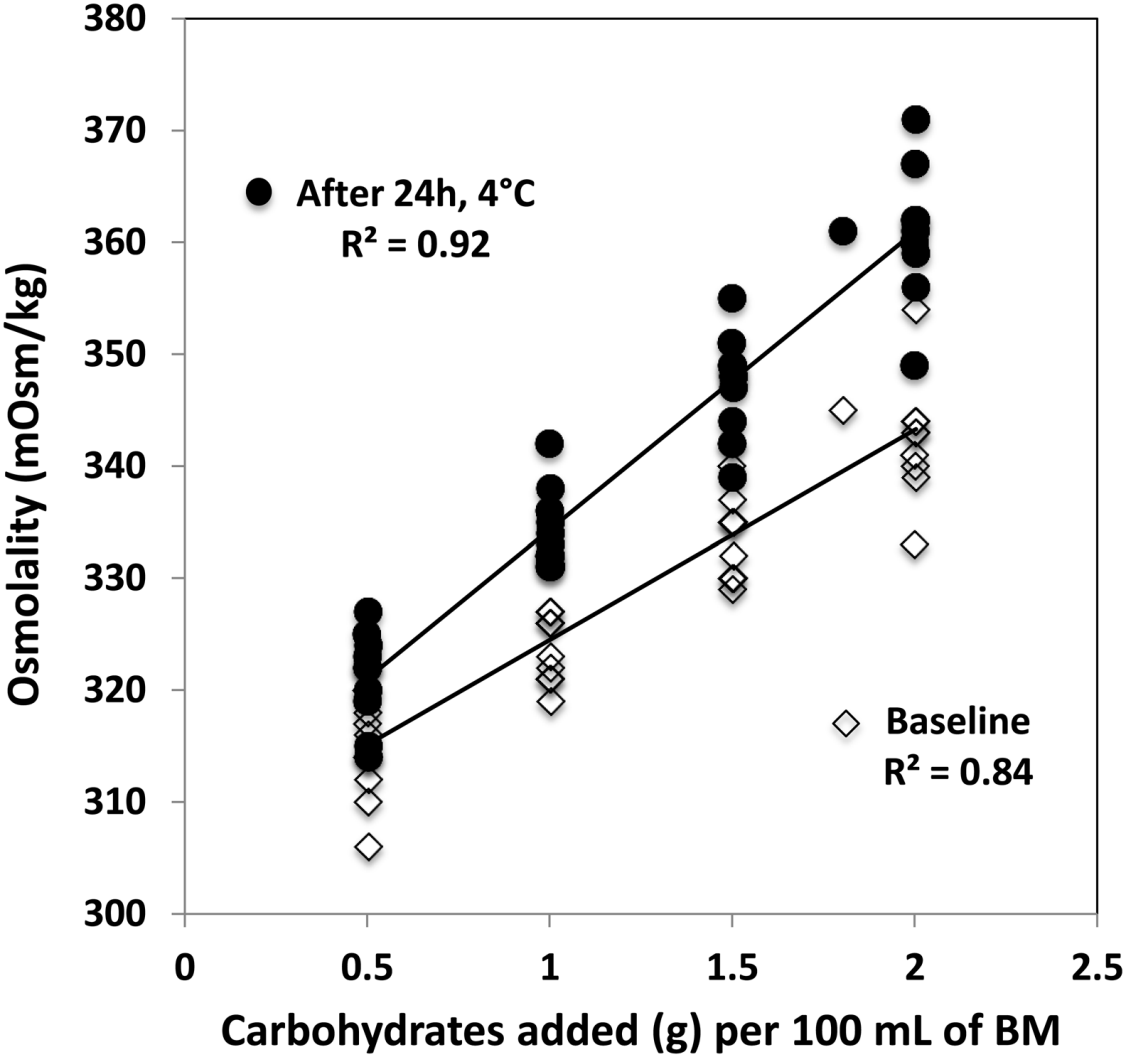
Osmolality change on fortified breast milk with carbohydrates after 24 hours of storage at 4°C. Diamond symbol represents baseline osmolality that measured immediate when breast milk was fortified and round symbol represents osmolality that measured after 24 hours storage at 4°C.

The osmolality prediction models for carbohydrate and protein fortifiers show significant linear correlation between the measured and predicted values. Coefficients of correlation for predictions of carbohydrates, whey-protein, hydrolyzed protein are R^2^ = 0.89, R^2^ = 0.65, R^2^ = 0.98, respectively (Fig 1). Following the addition of 1 g of carbohydrates, 1 g of whey protein, or 1 g of hydrolyzed protein per 100 mL breast milk, the average increase in osmolality was 20, 4, and 38 mOsm/kg respectively. The validation of prediction models for carbohydrates, whey-protein, hydrolyzed protein shows R^2^ = 0.76, R^2^ = 0.82, R^2^ = 0.99 (Fig 2). In contrast, addition of fat decreases osmolality minimally. The correlation between amount of fat added and the level of osmolality is weak (R^2^ = 0.49 and R^2^ = 0.33 for the prediction and the validation model, respectively). Further validation testing on the combinations of macronutrients depicts that the measured and predicted osmolality are also significantly correlated with R^2^ = 0.93 (Fig 3). To test the feasibility of the developed prediction model in clinical data (n = 696), the mean measured osmolality of breast milk samples is 299 ± 6 mOsm/kg and for target fortified breast milk samples is 437 ± 12 mOsm/kg. The two HMFs used for the validation increase osmolality by 106 ± 4 mOsm/kg (HMF1) and 56 ± 6 mOsm/kg (HMF2). The prediction model estimates mean osmolality of target fortified breast milk samples as 434 ± 9 mOsm/kg. Fig 4 shows the difference of measured and predicted osmolalities versus the range of predicted osmolalities. The average difference is 3 ± 11 mOsm/kg and is not statistically significantly different from zero. The osmolality of breast milk containing additional carbohydrates after 24 hours of storage in 4°C increases by 4% on average, as seen in Fig 5 The greater the amount of carbohydrates added to the breast milk, the greater the difference of osmolality values after 24 hours of storage.

## Discussion

The present study systematically investigates the impact of commonly used macronutrient supplements and human milk fortifiers on the osmolality of standard and individually fortified breast milk. We were able to predict the osmolality of milk feeds after adding combinations of macronutrient supplements with acceptable accuracy and precision. Our prediction equation showed a high validity in a clinical setting with almost 700 samples of analyzed target fortified breast milk.

Clinically, the prediction model will help clinicians and nutritionists in monitoring the osmolality level of feeds after target fortification. NICUs without the capability to measure osmolalities as a point of care (POC) can apply the equation to ensure that the final osmolality of individually prescribed fortification stays within accepted limits. NICUs with a POC option for osmolalities can use this formula as a quality control for human factors, (e.g. mixing errors) in order to prevent milk prescriptions where osmolality exceeds acceptable levels.

Our data shows that human milk fortifiers and carbohydrate supplement are the main contributors to the total osmolality of fortified breast milk batches. This increase may be caused by amylase activity of breast milk [10,14] Through amylase digestion, polysaccharides in carbohydrate fortified breast milk are broken down into their constituent mono- and oligosaccharides, increasing the absolute solute load and thus osmolality [10]. Further, we found that the storage of breast milk samples fortified with additional carbohydrates at 4°C for 24 hours significantly increased osmolality. This is confirmed by previous studies, which reported that storage at 4°C for 24 hours increased osmolality of fortified breast milk by 4% [10,11,15]. Although statistically significant, such changes do not appear clinically relevant because the final osmolality does not shift above unsafe ranges in most of the batches [15,16].

The second contributor to osmolality is protein. In our study, two protein supplements were tested. Their impact on final osmolality was found to be different, because the proteins are of different composition and nature: one product is made of whey protein isolate whereas the other is composed of hydrolyzed protein. We also observed a stark contrast in solubility between the two protein supplements, which may be attributed to variations in mineral composition [17,18].

Alternatively, supplementing milk with fat emulsion lowered the osmolality of breast milk. This finding can be considered to be a dilution effect as the product is specified as a 50% fat in water solution. Our findings are in line with those of a previous study, which showed that fat emulsions generally contribute little to osmolality; and in parenteral nutrition, formulations usually reduce total osmolality [19].

Final osmolality of milk feeds is a well-respected safety parameter in infant nutrition. Current guidelines suggest 450 mOsm/kg (i.e. osmolarity of 400 mOsm/L) as an upper limit of feeding osmolality to reduce feeding intolerance and the risk of necrotizing enterocolitis (NEC). However, the rationale for this number is based on one single publication in 1975. This study reported a high NEC rate in term infants after use of an elemental formula with an osmolality of 650 mOsm/L compared to a standard formula with 359mOsm/L [20]. The suggestion for the upper limit of feeding osmolality was chosen somewhere halfway between these two numbers, which is not very systematic and not supported by sound scientific evidence. Pearson *et al*. recently summarized its evolution from a single observation to becoming the basis for an over 40-year-old recommendation that still has a major impact on the development of infant dietary products and associated regulations [9]. Rochow *et al*. reported in their pilot trial on target fortification that 14% of breast milk batches had an osmolality between 450 and 480 mOsm/kg with none exceeding 480 mOsm/kg. There was no feeding intolerance observed during the intervention period [3]. Other studies have reported that the common NICU practice to add adjuvant medication and dietary products to expressed breast milk will increase final osmolality over 1000 mOsm/kg [21,22]. With this background, there is no scientific evidence that an osmolality up to 480 mOsm/kg is detrimental or may increase the NEC risk.

Our study was conducted using forty milk samples collected at different time points from one mother of a preterm infant. The average osmolality of all breast milk samples was 306 ± 8 mOsm/kg [(286, 319) (min, max)], which is within the osmolality range (median: 300 mOsm/kg) for fresh breast milk (n = 19) for mothers of preterm infants [15]. The variation of osmolality between our forty samples indicates that the composition of these samples is different. Moreover, as previously shown by Polberger, the intraindividual variation of macronutrient contents within the same mother is in the interindividual range of variation between mothers [2]. This has also been confirmed by our most recent data [3, 23]. We therefore consider our sample set as appropriate for this study.

A limitation of this study is that the developed prediction model is product-specific and cannot be transferred to other products used in NICUs. The linear equation must be established for each product and these equations can be used together for any combination of macronutrients to predict final osmolality. The key message is that the final osmolality has a linear relationship to the amount of macronutrients added.

This approach can also be used for quality control to identify samples suffering from improper mixing technique or accidental failure of fortification with nutrition supplementations. We would like to recall that in our pilot study of target fortification on breast milk, comparing observed and expected osmolality for any given sample, we were able to identify a 5% error rate in milk preparation [3]. This triggered a quality improvement initiative and a subsequent drop in the error rate. Since preterm infants have a low tolerance for such errors, checking osmolality after preparing target fortified breast milk would be appropriate to assure proper quality of feeds. As a related note, the osmolality added by each commercially available product varies; therefore, it is important to carefully select the nutrition supplements introduced to NICUs.

In conclusion, our study shows that the osmolality of target fortified breast milk samples can be reliably predicted in clinical routine and be used to enhance the safety of the fortification process.

## Supporting Information

S1 Table Study raw data containing increase in osmolality and added amount of each macronutrient in 100 mL of breast milk. (PDF)

S2 Table Study raw data containing measured osmolality increase (i.e. measurements using a freezing point device) and predicted osmolality increase (i.e. calculations from the prediction equations) on each macronutrient for a validation 1. (PDF)

S3 Table Study raw data containing measured (i.e. measurements using a freezing point device) and predicted (i.e. calculations from the prediction equations) osmolality of breast milk with added combinations of macronutrients for a validation 2. (PDF)

S4 Table Study raw data containing the measured and predicted osmolality using clinical samples from a previous target fortification study. (PDF)

S5 Table Study raw data containing osmolality changes on fortified breast milk with carbohydrates after 24 hours of storage at 4°C. (PDF)
