# Supplementary material for: Target Fortification of Breast Milk: Predicting the Final Osmolality of the Feeds
Source: PLoS One. 2016 Feb 10;11(2):e0148941. doi: 10.1371/journal.pone.0148941 (PMC4749227; doi:10.1371/journal.pone.0148941)
Supplement: S1 Table — (PDF) [file pone.0148941.s001.pdf]

S1 Table. Prediction: linear correlation between increase in osmolality and added amount of each macronutrient in 100 mL of breast milk. Graphs on carbohydrates (glucose polymer), protein 1(whey protein), protein 2 (hydrolyzed protein), and fat

Prediction model							
Polycose g/100mL	diff_Osmo mOsm/kg	Beneprotein (protein 1) g/100mL	diff_Osmo mOsm/kg	Aptamil/Protein2 g/100mL	diff_Osmo mOsm/kg	lipid ml/100mL	diff_Osmo mOsm/kg
0.501	14	0.201	-4	0.201	10	0.5	3
0.503	10	0.224	0	0.209	11	0.5	0
0.502	12	0.214	-1	0.193	8	0.5	1
0.502	6	0.210	0	0.214	6	0.5	1
0.500	8	0.207	0	0.184	6	0.5	1
0.504	9	0.226	3	0.213	8	0.5	0
0.503	12	0.212	0	0.212	6	0.5	4
0.501	13	0.225	4	0.203	2	0.5	2
0.499	14	0.197	-2	0.198	7	0.5	2
0.502	4	0.202	-1	0.197	13	0.5	-1
1.002	23	0.678	2	0.498	19.5	1	0
1.000	22	0.703	6	0.515	24	1	0
1.002	15	0.515	3	0.497	18	1	0
1.002	19	0.510	0	0.518	17	1	0
1.002	16	0.508	0	0.499	15	1	-1
1.000	18	0.527	3	0.540	18	1	0
1.003	25	0.515	0	0.511	20	1	2
1.002	18	0.527	2	0.507	16	1	0
0.999	26	0.496	-1	0.492	16	1	1
1.000	15	0.499	-5	0.489	21	1	-4
1.501	34	1.183	6	0.952	42	2	0
1.500	26	1.203	7	1.018	41	2	-3
1.501	29	1.016	6	0.991		2	1
1.503	31	1.014	1	1.018	37	2	-1
1.503	26	1.005	4	1.000	32	2	-2
1.503	27	1.031	7	1.050	40	2	-1
1.500	35	1.014	2	1.010	40	2	1
1.500	26	1.031	4	1.009	32	2	0
1.499	36	0.996	2	1.003	36	2	0
1.498	28	0.991	-2	1.008	40	2	-2
2.003	41	2.173	8	2.004	74	4	0
1.999	37	2.198	12	2.005	81	4	-4
2.001	38	2.023	9	1.983	70	4	-1
2.001	36	2.034	7	2.018	79	4	-4
2.002	37	2.041	6	2.031	75	4	-4
2.003	36	2.048	10	2.078	80	4	-1
1.999	39	2.018	6	2.035	81	4	-4
2.003	34	2.030	15	2.030	70	4	-3
2.003	48	1.997	9	2.059	73	4	-4
1.804	33	1.588	3	2.007	82	4	-3
