# Supplementary material for: Target Fortification of Breast Milk: Predicting the Final Osmolality of the Feeds
Source: PLoS One. 2016 Feb 10;11(2):e0148941. doi: 10.1371/journal.pone.0148941 (PMC4749227; doi:10.1371/journal.pone.0148941)
Supplement: S2 Table — (PDF) [file pone.0148941.s002.pdf]

**S2 Table.** Validation 1: correlation between measured osmolality increase (i.e. measurements using a freezing point device) and predicted osmolality increase (i.e. calculations from the prediction equations) on single macronutrient. Graphs on carbohydrates (glucose polymer), protein 1 (whey protein), protein 2 (hydrolyzed protein), and fat

Validation on prediction

| Polycose g/100mL | Measured osmolality increase mOsm/kg | Predicted osmolality increase mOsm/kg |
|---|---|---|
| 0.800 | 17 | 16 |
| 0.803 | 13 | 16 |
| 0.802 | 20 | 16 |
| 0.814 | 19 | 16 |
| 0.798 | 7 | 16 |
| 1.596 | 32 | 31 |
| 1.604 | 26 | 31 |
| 1.604 | 31 | 31 |
| 1.604 | 37 | 31 |
| 1.603 | 27 | 31 |

| Beneprotein (Protein 1) g/100mL | Measured osmolality increase mOsm/kg | Predicted osmolality increase mOsm/kg |
|---|---|---|
| 0.396 | 1 | 0 |
| 0.403 | 1 | 0 |
| 0.393 | 1 | 0 |
| 0.402 | 4 | 0 |
| 0.393 | 2 | 0 |
| 1.632 | 8 | 7 |
| 1.619 | 7 | 7 |
| 1.617 | 9 | 7 |
| 1.586 | 12 | 6 |
| 1.573 | 7 | 6 |

| Aptamil/Protein 2 g/100mL | Measured osmolality increase mOsm/kg | Predicted osmolality increase mOsm/kg |
|---|---|---|
| 0.467 | 19 | 17 |
| 0.410 | 14 | 15 |
| 0.401 | 15 | 15 |
| 0.404 | 14 | 15 |
| 0.408 | 19 | 15 |
| 1.652 | 65 | 62 |
| 1.580 | 60 | 60 |
| 1.600 | 58 | 60 |
| 1.609 | 59 | 61 |
| 1.636 | 57 | 62 |

| Lipid ml/100mL | Measured osmolality increase mOsm/kg | Predicted osmolality increase mOsm/kg |
|---|---|---|
| 0.8 | 5 | 1 |
| 0.8 | -1 | 1 |
| 0.8 | -3 | 1 |
| 0.8 | 4 | 1 |
| 0.8 | -2 | 1 |
| 3.2 | -1 | -2 |
| 3.2 | -3 | -2 |
| 3.2 | -4 | -2 |
| 3.2 | -4 | -2 |
| 3.2 | -2 | -2 |
