# Supplementary material for: Target Fortification of Breast Milk: Predicting the Final Osmolality of the Feeds
Source: PLoS One. 2016 Feb 10;11(2):e0148941. doi: 10.1371/journal.pone.0148941 (PMC4749227; doi:10.1371/journal.pone.0148941)
Supplement: S3 Table — (PDF) [file pone.0148941.s003.pdf]

S3 Table. Validation 2: correlation between measured (i.e. measurements using a freezing point device) and predicted (i.e. calculations from the prediction equations) osmolality of breast milk with added combinations of macronutrients

	Fat mL/100mL	Protein g/100mL	Carbohydrates g/100mL	Measured osmolality mOsm/kg	Predicted osmolality mOsm/kg	after 24h mOsm/kg
Milk 1	0.98			403	414	429
Milk 2	2.36			408	415	432
Milk 3	3.22			402	407	424
Milk 4	0.36			404	413	436
Milk 5	2.24			404	402	431
Milk 6	0.00			359	365	393
Milk 7	0.66			357	367	377
Milk 8	2.18			353	358	376
Milk 9	0.00			362	364	378
Milk 10	0.46			351	354	378
Milk 1		1.21		419	421	442
Milk 2		1.31		411	424	428
Milk 3		1.30		423	417	444
Milk 4		0.79		404	418	434
Milk 5		1.09		404	410	435
Milk 6		1.33		365	371	394
Milk 7		1.49		369	375	387
Milk 8		1.50		361	368	380
Milk 9		0.57		361	367	386
Milk 10		1.13		358	360	383
Milk 1			1.09	433	435	466
Milk 2			1.13	434	439	464
Milk 3			1.33	424	436	461
Milk 4			1.53	444	443	475
Milk 5			1.36	417	430	466
Milk 6			2.11	394	404	452
Milk 7			2.14	407	408	444
Milk 8			2.31	391	404	442
Milk 9			2.72	394	415	448
Milk 10			2.34	382	399	439
Milk 1	0.98	1.20		411	420	441
Milk 2	2.36	1.29		405	422	426
Milk 3	3.22	1.34		403	414	429
Milk 4	0.36	0.79		414	417	438
Milk 5	2.24	1.06		394	408	419
Milk 6	0.00	1.33		365	371	394
Milk 7	0.66	1.50		363	375	384
Milk 8	2.18	1.51		361	366	380
Milk 9	0.00	0.57		361	367	386
Milk 10	0.46	1.13		360	360	385
Milk 1	0.98		1.10	429	434	468
Milk 2	2.36		1.15	427	437	464
Milk 3	3.22		1.32	432	432	464
Milk 4	0.36		1.55	429	442	471
Milk 5	2.24		1.34	408	428	448
Milk 6	0.00		2.11	394	404	452
Milk 7	0.66		2.14	400	407	446
Milk 8	2.18		2.29	373	402	413
Milk 9	0.00		2.72	394	415	448
Milk 10	0.46		2.36	389	399	442
Milk 1		1.19	1.10	446	442	485
Milk 2		1.29	1.15	456	446	486
Milk 3		1.32	1.34	437	443	469
Milk 4		0.79	1.55	456	447	490
Milk 5		1.09	1.34	437	435	472
Milk 6		1.34	2.09	403	411	456
Milk 7		1.49	2.14	413	416	448
Milk 8		1.50	2.32	400	412	447
Milk 9		0.57	2.55	414	415	455
Milk 10		1.12	2.32	388	404	435
Milk 1	0.98	1.20	1.11	442	441	480
Milk 2	2.36	1.30	1.14	429	443	454
Milk 3	3.22	1.31	1.34	425	439	466
Milk 4	0.36	0.79	1.54	439	446	480
Milk 5	2.24	1.09	1.31	416	433	452
Milk 6	0.00	1.34	2.09	403	411	456
Milk 7	0.66	1.52	2.13	403	415	445
Milk 8	2.18	1.49	2.34	393	410	439
Milk 9	0.00	0.57	2.55	414	415	455
Milk 10	0.46	1.10	2.33	395	404	447
