# Supplementary material for: Target Fortification of Breast Milk: Predicting the Final Osmolality of the Feeds
Source: PLoS One. 2016 Feb 10;11(2):e0148941. doi: 10.1371/journal.pone.0148941 (PMC4749227; doi:10.1371/journal.pone.0148941)
Supplement: S4 Table — (PDF) [file pone.0148941.s004.pdf]

S4 Table. Deviation between measured & predicted osmolality, using clinical samples from the pilot target fortification (TFO) study (n=696)

sample#	measured osmolality mOsm/kg	calculated osmolality mOsm/kg	(measured-calculated) osmolality mOsm/kg
1	400	367	33
2	425	392	33
3	408	400	8
4	436	400	36
5	405	403	2
6	414	405	9
7	421	409	12
8	418	409	9
9	409	412	-3
10	450	412	38
11	422	413	9
12	400	413	-13
13	419	414	5
14	410	415	-5
15	413	416	-3
16	433	416	17
17	405	416	-11
18	404	416	-12
19	416	417	-1
20	421	417	4
21	423	418	5
22	425	418	7
23	436	418	18
24	436	419	17
25	424	419	5
26	415	419	-4
27	427	419	8
28	412	419	-7
29	418	420	-2
30	433	420	13
31	440	420	20
32	428	420	8
33	429	420	9
34	451	420	31
35	436	421	15
36	422	421	1
37	412	421	-9
38	410	421	-11
39	422	421	1
40	424	421	3
41	415	421	-6
42	437	422	15
43	423	422	1
44	426	422	4
45	414	422	-8
46	428	422	6
47	421	422	-1
48	416	422	-6
49	440	422	18
50	420	422	-2
51	424	423	1
52	428	423	5
53	414	423	-9

sample#	measured osmolality	calculated osmolality	(measured-calculated) osmolality
54	421	423	-2
55	421	423	-2
56	437	423	14
57	421	423	-2
58	429	423	6
59	418	424	-6
60	415	424	-9
61	398	424	-26
62	431	424	7
63	414	424	-10
64	433	424	9
65	430	424	6
66	430	424	6
67	440	424	16
68	435	424	11
69	452	425	27
70	423	425	-2
71	427	425	2
72	425	425	0
73	435	425	10
74	409	425	-16
75	409	425	-16
76	431	425	6
77	428	425	3
78	426	425	1
79	424	425	-1
80	451	425	26
81	423	425	-2
82	420	425	-5
83	423	425	-2
84	422	425	-3
85	419	426	-7
86	446	426	20
87	442	426	16
88	426	426	0
89	410	426	-16
90	434	426	8
91	429	426	3
92	426	426	0
93	429	426	3
94	439	426	13
95	410	426	-16
96	437	426	11
97	399	426	-27
98	443	426	17
99	412	426	-14
100	430	426	4
101	425	426	-1
102	419	426	-7
103	434	426	8
104	427	427	0
105	432	427	5
106	422	427	-5
107	429	427	2
108	440	427	13
109	426	427	-1
110	439	427	12
111	440	427	13
112	425	427	-2

sample#	measured osmolality	calculated osmolality	(measured-calculated) osmolality
113	427	427	0
114	424	427	-3
115	442	427	15
116	428	427	1
117	428	427	1
118	436	427	9
119	441	427	14
120	420	427	-7
121	427	427	0
122	442	427	15
123	411	427	-16
124	440	427	13
125	414	427	-13
126	439	428	11
127	432	428	4
128	443	428	15
129	412	428	-16
130	443	428	15
131	430	428	2
132	437	428	9
133	449	428	21
134	424	428	-4
135	432	428	4
136	437	428	9
137	429	428	1
138	435	428	7
139	434	428	6
140	436	428	8
141	422	428	-6
142	430	428	2
143	415	428	-13
144	412	428	-16
145	442	428	14
146	421	428	-7
147	421	428	-7
148	435	428	7
149	428	428	0
150	412	428	-16
151	433	428	5
152	424	428	-4
153	435	429	6
154	438	429	9
155	418	429	-11
156	439	429	10
157	432	429	3
158	429	429	0
159	431	429	2
160	430	429	1
161	433	429	4
162	432	429	3
163	441	429	12
164	418	429	-11
165	408	429	-21
166	439	429	10
167	432	429	3
168	443	429	14
169	428	429	-1
170	440	429	11
171	426	429	-3

sample#	measured osmolality	calculated osmolality	(measured-calculated) osmolality
172	449	429	20
173	437	429	8
174	403	429	-26
175	446	429	17
176	438	429	9
177	425	429	-4
178	417	429	-12
179	437	429	8
180	428	429	-1
181	438	429	9
182	420	429	-9
183	427	429	-2
184	434	430	4
185	430	430	0
186	437	430	7
187	436	430	6
188	429	430	-1
189	438	430	8
190	413	430	-17
191	426	430	-4
192	424	430	-6
193	443	430	13
194	432	430	2
195	433	430	3
196	436	430	6
197	438	430	8
198	431	430	1
199	410	430	-20
200	421	430	-9
201	425	430	-5
202	447	430	17
203	456	430	26
204	426	430	-4
205	410	430	-20
206	442	430	12
207	422	430	-8
208	438	430	8
209	443	430	13
210	451	430	21
211	436	430	6
212	429	430	-1
213	438	430	8
214	434	430	4
215	443	430	13
216	438	430	8
217	433	430	3
218	437	430	7
219	447	430	17
220	420	430	-10
221	422	430	-8
222	437	430	7
223	438	430	8
224	442	430	12
225	435	430	5
226	437	430	7
227	425	430	-5
228	446	431	15
229	428	431	-3
230	422	431	-9

sample#	measured osmolality	calculated osmolality	(measured-calculated) osmolality
231	425	431	-6
232	435	431	4
233	428	431	-3
234	439	431	8
235	436	431	5
236	435	431	4
237	440	431	9
238	424	431	-7
239	435	431	4
240	423	431	-8
241	431	431	0
242	436	431	5
243	442	431	11
244	431	431	0
245	436	431	5
246	442	431	11
247	441	431	10
248	447	431	16
249	432	431	1
250	443	431	12
251	441	431	10
252	442	431	11
253	447	431	16
254	444	432	12
255	451	432	19
256	445	432	13
257	434	432	2
258	442	432	10
259	439	432	7
260	460	432	28
261	424	432	-8
262	445	432	13
263	444	432	12
264	434	432	2
265	436	432	4
266	415	432	-17
267	431	432	-1
268	440	432	8
269	421	432	-11
270	445	432	13
271	435	432	3
272	437	432	5
273	452	432	20
274	440	432	8
275	449	432	17
276	443	432	11
277	437	432	5
278	426	432	-6
279	437	432	5
280	443	432	11
281	431	432	-1
282	446	432	14
283	412	432	-20
284	434	432	2
285	457	432	25
286	431	432	-1
287	418	432	-14
288	436	432	4
289	435	432	3

sample#	measured osmolality	calculated osmolality	(measured-calculated) osmolality
290	441	433	8
291	447	433	14
292	443	433	10
293	436	433	3
294	429	433	-4
295	428	433	-5
296	448	433	15
297	426	433	-7
298	437	433	4
299	445	433	12
300	440	433	7
301	432	433	-1
302	434	433	1
303	439	433	6
304	441	433	8
305	430	433	-3
306	425	433	-8
307	436	433	3
308	418	433	-15
309	438	433	5
310	443	433	10
311	435	433	2
312	426	433	-7
313	439	433	6
314	445	433	12
315	441	433	8
316	434	433	1
317	441	433	8
318	440	433	7
319	447	433	14
320	439	433	6
321	418	433	-15
322	440	433	7
323	435	433	2
324	436	433	3
325	431	434	-3
326	438	434	4
327	443	434	9
328	440	434	6
329	455	434	21
330	439	434	5
331	443	434	9
332	440	434	6
333	448	434	14
334	438	434	4
335	431	434	-3
336	426	434	-8
337	446	434	12
338	436	434	2
339	428	434	-6
340	435	434	1
341	428	434	-6
342	429	434	-5
343	431	434	-3
344	462	434	28
345	445	434	11
346	438	434	4
347	430	434	-4
348	427	434	-7

sample#	measured osmolality	calculated osmolality	(measured-calculated) osmolality
349	433	434	-1
350	429	434	-5
351	439	434	5
352	458	434	24
353	440	434	6
354	443	434	9
355	435	434	1
356	443	434	9
357	442	434	8
358	439	434	5
359	426	435	-9
360	447	435	12
361	435	435	0
362	437	435	2
363	432	435	-3
364	428	435	-7
365	440	435	5
366	442	435	7
367	477	435	42
368	453	435	18
369	441	435	6
370	434	435	-1
371	438	435	3
372	432	435	-3
373	410	435	-25
374	435	435	0
375	442	435	7
376	427	435	-8
377	446	435	11
378	436	435	1
379	440	435	5
380	442	435	7
381	462	435	27
382	419	435	-16
383	432	435	-3
384	443	435	8
385	449	435	14
386	450	435	15
387	435	435	0
388	441	435	6
389	441	435	6
390	434	435	-1
391	455	435	20
392	438	435	3
393	430	435	-5
394	447	436	11
395	456	436	20
396	451	436	15
397	438	436	2
398	447	436	11
399	432	436	-4
400	457	436	21
401	421	436	-15
402	453	436	17
403	439	436	3
404	438	436	2
405	445	436	9
406	441	436	5
407	438	436	2

sample#	measured osmolality	calculated osmolality	(measured-calculated) osmolality
408	444	436	8
409	411	436	-25
410	436	436	0
411	428	436	-8
412	434	436	-2
413	405	436	-31
414	432	436	-4
415	439	436	3
416	470	436	34
417	450	436	14
418	438	436	2
419	455	436	19
420	440	436	4
421	444	436	8
422	436	436	0
423	437	436	1
424	426	436	-10
425	430	436	-6
426	442	436	6
427	440	436	4
428	444	436	8
429	453	436	17
430	423	437	-14
431	435	437	-2
432	436	437	-1
433	452	437	15
434	458	437	21
435	439	437	2
436	441	437	4
437	433	437	-4
438	442	437	5
439	451	437	14
440	446	437	9
441	455	437	18
442	441	437	4
443	451	437	14
444	426	437	-11
445	449	437	12
446	442	437	5
447	439	437	2
448	433	437	-4
449	437	437	0
450	439	437	2
451	430	437	-7
452	446	437	9
453	436	437	-1
454	446	437	9
455	447	437	10
456	431	437	-6
457	441	437	4
458	440	437	3
459	450	437	13
460	429	437	-8
461	420	437	-17
462	437	437	0
463	427	437	-10
464	436	437	-1
465	438	437	1
466	456	437	19

sample#	measured osmolality	calculated osmolality	(measured-calculated) osmolality
467	435	437	-2
468	442	437	5
469	447	437	10
470	445	437	8
471	441	437	4
472	462	437	25
473	457	437	20
474	435	438	-3
475	417	438	-21
476	438	438	0
477	441	438	3
478	443	438	5
479	452	438	14
480	444	438	6
481	446	438	8
482	426	438	-12
483	453	438	15
484	427	438	-11
485	443	438	5
486	420	438	-18
487	413	438	-25
488	422	438	-16
489	437	438	-1
490	452	438	14
491	439	438	1
492	428	438	-10
493	435	438	-3
494	431	438	-7
495	422	438	-16
496	423	438	-15
497	465	438	27
498	439	438	1
499	442	438	4
500	439	438	1
501	440	438	2
502	432	438	-6
503	458	439	19
504	435	439	-4
505	449	439	10
506	442	439	3
507	446	439	7
508	430	439	-9
509	432	439	-7
510	443	439	4
511	436	439	-3
512	442	439	3
513	451	439	12
514	429	439	-10
515	444	439	5
516	438	439	-1
517	434	439	-5
518	434	439	-5
519	438	439	-1
520	438	439	-1
521	441	439	2
522	447	439	8
523	415	439	-24
524	441	439	2
525	441	439	2

sample#	measured osmolality	calculated osmolality	(measured-calculated) osmolality
526	427	439	-12
527	458	439	19
528	434	439	-5
529	446	439	7
530	448	439	9
531	438	439	-1
532	432	439	-7
533	430	439	-9
534	452	439	13
535	427	439	-12
536	441	439	2
537	453	439	14
538	440	439	1
539	445	439	6
540	434	439	-5
541	449	440	9
542	419	440	-21
543	426	440	-14
544	436	440	-4
545	420	440	-20
546	424	440	-16
547	442	440	2
548	433	440	-7
549	440	440	0
550	450	440	10
551	454	440	14
552	455	440	15
553	436	440	-4
554	440	440	0
555	435	440	-5
556	442	440	2
557	441	440	1
558	430	440	-10
559	439	440	-1
560	443	440	3
561	434	440	-6
562	428	440	-12
563	454	440	14
564	445	440	5
565	449	440	9
566	442	440	2
567	428	441	-13
568	462	441	21
569	459	441	18
570	435	441	-6
571	441	441	0
572	449	441	8
573	443	441	2
574	442	441	1
575	451	441	10
576	433	441	-8
577	461	441	20
578	443	441	2
579	436	441	-5
580	472	441	31
581	440	441	-1
582	434	441	-7
583	453	441	12
584	446	441	5

sample#	measured osmolality	calculated osmolality	(measured-calculated) osmolality
585	453	442	11
586	440	442	-2
587	435	442	-7
588	443	442	1
589	445	442	3
590	396	442	-46
591	449	442	7
592	440	442	-2
593	445	442	3
594	411	442	-31
595	444	442	2
596	436	442	-6
597	430	442	-12
598	451	442	9
599	449	442	7
600	457	442	15
601	457	442	15
602	441	443	-2
603	467	443	24
604	472	443	29
605	444	443	1
606	450	443	7
607	445	443	2
608	446	443	3
609	446	443	3
610	448	443	5
611	426	443	-17
612	451	443	8
613	444	443	1
614	445	443	2
615	428	443	-15
616	464	443	21
617	453	443	10
618	452	443	9
619	442	443	-1
620	434	443	-9
621	431	443	-12
622	440	444	-4
623	449	444	5
624	440	444	-4
625	419	444	-25
626	452	444	8
627	441	444	-3
628	443	444	-1
629	441	444	-3
630	453	444	9
631	477	444	33
632	443	445	-2
633	449	445	4
634	445	445	0
635	439	445	-6
636	468	445	23
637	451	445	6
638	441	445	-4
639	419	445	-26
640	447	445	2
641	454	445	9
642	448	445	3
643	459	445	14

sample#	measured osmolality	calculated osmolality	(measured-calculated) osmolality
644	457	445	12
645	453	445	8
646	456	445	11
647	408	445	-37
648	453	445	8
649	465	445	20
650	452	445	7
651	460	445	15
652	460	445	15
653	469	446	23
654	455	446	9
655	463	446	17
656	445	446	-1
657	454	446	8
658	440	446	-6
659	444	446	-2
660	463	446	17
661	453	447	6
662	447	447	0
663	431	447	-16
664	439	447	-8
665	471	447	24
666	459	447	12
667	443	447	-4
668	443	448	-5
669	456	448	8
670	447	449	-2
671	460	449	11
672	445	449	-4
673	446	449	-3
674	447	449	-2
675	435	450	-15
676	476	450	26
677	460	451	9
678	441	451	-10
679	440	451	-11
680	435	451	-16
681	470	451	19
682	456	451	5
683	445	452	-7
684	440	452	-12
685	466	452	14
686	465	452	13
687	442	452	-10
688	436	453	-17
689	433	454	-21
690	469	454	15
691	475	454	21
692	468	455	13
693	460	455	5
694	463	464	-1
695	436	474	-38
696	442	475	-33
