# Supplementary material for: Target Fortification of Breast Milk: Predicting the Final Osmolality of the Feeds
Source: PLoS One. 2016 Feb 10;11(2):e0148941. doi: 10.1371/journal.pone.0148941 (PMC4749227; doi:10.1371/journal.pone.0148941)
Supplement: S5 Table — (PDF) [file pone.0148941.s005.pdf]

S5 Table. Osmolality change on fortified breast milk with carbohydrates (24h, 4 °C)

Polycose g/100mL	Osmolality,measured immediately after mixing mOsm/kg	Osmolality after storage (24h, 4 °C) mOsm/kg
0.5008	317	319
0.5032	314	315
0.5016	318	322
0.5020	310	320
0.5004	314	322
0.5036	312	324
0.5032	306	314
0.5008	322	323
0.4988	320	325
0.5024	316	327
1.0024	326	332
1.0004	326	331
1.0020	321	334
1.0016	323	333
1.0016	322	338
1.0004	321	332
1.0028	319	331
1.0024	327	335
0.9992	332	342
1.0000	327	336
1.5008	337	344
1.4996	330	342
1.5012	335	349
1.5032	335	348
1.5032	332	348
1.5032	330	347
1.5004	329	339
1.5000	335	349
1.4992	342	355
1.4984	340	351
2.0028	344	359
1.9992	341	360
2.0012	344	367
2.0008	340	362
2.0024	343	361
2.0028	339	356
1.9992	333	349
2.0032	343	362
2.0028	354	371
1.8036	345	361
